# Chronic ultraviolet irradiation induces memory deficits via dysregulation of the dopamine pathway

**DOI:** 10.1038/s12276-024-01242-x

**Published:** 2024-06-03

**Authors:** Kyeong-No Yoon, Sun Yong Kim, Jungeun Ji, Yidan Cui, Qing‐Ling Quan, Gunhyuk Park, Jang-Hee Oh, Ji Su Lee, Joon-Yong An, Jin Ho Chung, Yong-Seok Lee, Dong Hun Lee

**Affiliations:** 1 Department of Biomedical Sciences, Seoul National University Graduate School, Seoul, Republic of Korea; 2 Laboratory of Cutaneous Aging Research, Biomedical Research Institute, Seoul National University Hospital, Seoul, Republic of Korea; 3 Institute of Human-Environmental Interface Biology, Medical Research Center, Seoul National University, Seoul, Republic of Korea; 4 Department of Physiology, Seoul National University College of Medicine, Seoul, Republic of Korea; 5 Department of Integrated Biomedical and Life Science, Korea University, Seoul, Republic of Korea; 6 BK21FOUR R&E Center for Learning Health Systems, Korea University, Seoul, Republic of Korea; 7 Department of Dermatology, Seoul National University Hospital, Seoul National University College of Medicine, Seoul, Republic of Korea; 8 Herbal Medicine Resources Research Center, Korea Institute of Oriental Medicine, Seoul, Republic of Korea; 9 School of Biosystem and Biomedical Science, College of Health Science, Korea University, Seoul, Republic of Korea; 10 Institute on Aging, Seoul National University, Seoul, Republic of Korea; 11 Neuroscience Research Institute, Seoul National University College of Medicine, Seoul, Republic of Korea; 12 Wide River Institute of Immunology, Seoul National University, Hongcheon, Republic of Korea

**Keywords:** Cognitive control, Cellular neuroscience

## Abstract

The effects of ultraviolet (UV) radiation on brain function have previously been investigated; however, the specific neurotransmitter-mediated mechanisms responsible for UV radiation-induced neurobehavioral changes remain elusive. In this study, we aimed to explore the mechanisms underlying UV radiation-induced neurobehavioral changes. In a mouse model, we observed that UV irradiation of the skin induces deficits in hippocampal memory, synaptic plasticity, and adult neurogenesis, as well as increased dopamine levels in the skin, adrenal glands, and brain. Chronic UV exposure altered the expression of genes involved in dopaminergic neuron differentiation. Furthermore, chronic peripheral dopamine treatments resulted in memory deficits. Systemic administration of a dopamine D1/D5 receptor antagonist reversed changes in memory, synaptic plasticity, adult neurogenesis, and gene expression in UV-irradiated mice. Our findings provide converging evidence that chronic UV exposure alters dopamine levels in the central nervous system and peripheral organs, including the skin, which may underlie the observed neurobehavioral shifts, such as hippocampal memory deficits and impaired neurogenesis. This study underscores the importance of protection from UV exposure and introduces the potential of pharmacological approaches targeting dopamine receptors to counteract the adverse neurological impacts of UV exposure.

## Unveiling dopamine’s role in UV-induced neurobehavioral changes

Exposure to ultraviolet radiation, which is a harmful type of light from the sun, can cause skin inflammation and other health problems. This study looked at how UV radiation affects the brain, specifically focusing on dopamine, a chemical in the brain that helps control movement and emotional responses. The researchers did experiments on mice, exposing them to UV radiation and observing changes in their behavior and brain function. They found that UV radiation increased dopamine levels in the skin, adrenal glands, and brain. This increase was linked to memory loss and changes in behavior. The researchers concluded that UV radiation can affect brain function and behavior by changing dopamine levels. This study shows the importance of protecting against UV radiation and suggests that targeting dopamine receptors could help reduce the negative brain effects of UV exposure.

This summary was initially drafted using artificial intelligence, then revised and fact-checked by the author.

## Introduction

Exposure to ultraviolet (UV) radiation can be transient or chronic in daily life. Transient UV exposure induces sunburn or skin inflammation, activates growth factor receptors, initiates subsequent signal transduction pathways, and releases cytokines. Chronic UV exposure can lead to chronic skin inflammation, immunosuppression, photocarcinogenesis, and photoaging^1,2^. Photoaging refers to premature skin aging caused by prolonged and excessive exposure to UV radiation, whether from the sun or artificial sources such as tanning beds^3^. UV exposure to the skin triggers skin-related biochemical responses and impacts signal transduction pathways in other organs. Furthermore, several studies have suggested that chronic UV exposure can alter the levels of hormones, proteins, and small molecules, such as melanocyte-stimulating hormone^4^, β-endorphin^5^, nitric oxide^6,7^, and urocanic acid^8^, in the skin or blood.

Interestingly, studies have shown that changes in UV-induced mediators in the blood may also affect the brain^5,8,9^. UV exposure elevates the levels of β-endorphin, which is transmitted through the blood to the brain in the skin of mice, resulting in opioid-related antinociception and ultimately leading to addiction to UV light in mice^5^. Moderate UV exposure can increase blood urocanic acid levels and enhance learning and memory in the mouse brain via the glutamate biosynthetic pathway^8^. The serum level of corticosterone is notably increased after UV exposure in mice, potentially contributing to the UV irradiation-induced decrease in hippocampal neurogenesis^9^. These findings suggest a close interconnection between UV-induced changes in mediators in the skin and bloodstream and brain function. Sophisticated communication between the skin and brain maintains and regulates homeostasis and plays a pivotal role in aging and age-related diseases^10^.

Neurotransmitters are crucial neuronal mediators involved in brain aging and disorders such as Parkinson’s Disease and Alzheimer’s disease^11^. Both the skin and neuroendocrine systems produce neurotransmitters under stimulating conditions^10,12–14^. Furthermore, neurotransmitters produced by the skin might influence specific brain areas that constitute the skin–brain axis^15^. For example, serotonin is a neurotransmitter generated in the skin that bridges the central and peripheral nervous systems, underscoring the close relationship between the nervous system and the skin^16–18^. Additionally, dopamine levels in the skin have been found to increase immediately after UV exposure^19^. Dopamine is instrumental in various brain functions and is commonly linked to feelings of pleasure, reward, motivation, and memory^20^. However, sustaining a balanced and regulated level of dopamine signaling in the brain is essential since excessive or dysregulated dopamine signaling can harm mental and physical health^21,22^. The biological implications of elevated dopamine levels in the blood, particularly regarding brain function, remain unexplored.

Neurotransmitters play pivotal roles in determining the status of the brain during disease and aging^11^. While mounting evidence suggests that UV exposure to the skin may lead to neurobehavioral changes in the brain, only a few plausible neuronal mediators, such as β-endorphin, have been explored. Hence, the molecular and cellular mechanisms underlying UV-induced neurobehavioral changes remain largely undiscovered. Based on all the available evidence, we hypothesize that neurotransmitters serve as potential conduits for skin–brain communication in UV-induced neurobehavioral shifts. Thus, we aimed to uncover the specific neurotransmitter-mediated mechanisms underlying UV-induced neurobehavioral changes and to investigate potential neurotransmitters in the serum and brain post-UV irradiation to identify candidate molecules.

## Materials and methods

### Animal experiments

Six-week-old female SKH-1 hairless mice were purchased from Orient Bio (Seongnam, South Korea). The animals were allowed *ad libitum* access to food and were acclimatized for one week before the study. All experimental protocols were approved by the Institutional Animal Care and Use Committee (No. 20–0271) of the Biomedical Research Institute at Seoul National University Hospital and were performed in accordance with the relevant guidelines and regulations. SKH-1 hairless mice were randomly allocated to two groups.

### UV irradiation

**Fig. 1:**
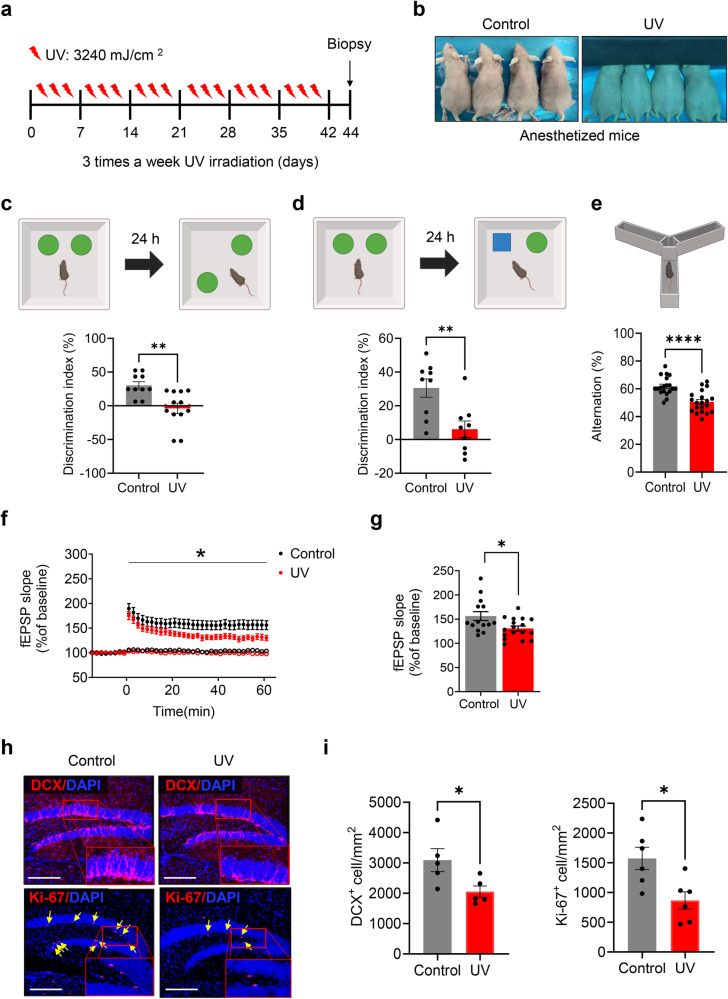
Effects of UV irradiation on memory function and hippocampal neurogenesis. **a** Overview of the experimental design. **b** Representative images of mice exposed to either sham irradiation (control) or UV irradiation (UV). **c**, **d** Object place recognition (OPR) and novel object recognition (NOR) tests. After 6 weeks of UV irradiation, the discrimination index was calculated by subtracting the time spent exploring a familiar object (F) from the time spent exploring a novel object (N), which is the difference between the total time spent exploring both objects. (discrimination index) = (N − F)/(N + F). OPR test: control group (*n* = 10), UV-irradiated group (*n* = 12); NOR test: control group (*n* = 9), UV-irradiated group (*n* = 9). Mann–Whitney *U* test, ^**^P < 0.01 vs. the control group. Each bar represents the mean ± SEM of each group. **e** Diagram representing the Y-maze test. To calculate the number of Y-maze alternations, the number of triads (sets of three consecutive arm entries) was determined, and the number of alternations between these triads was counted. An alternation was defined as visiting all three arms of a triad without revisiting the previously visited arm. Y-maze alternation = (number of alternations/number of triads − 2) × 100. Control group (*n* = 19), UV-irradiated group (*n* = 20) Mann-Whitney *U* test, ^****^P < 0.0001 vs. the control group. Each bar represents the mean ± SEM of each group. **f** Time course of the fEPSP slope representing LTP of the 6-week UV-treated mice and the control mice (control, *n* = 14 from nine mice; UV-irradiated, *n* = 17 from 8 mice; one-way analysis of variance, effect of UV treatment, ^*^P < 0.05). **g** Average fEPSP slope 51–60 min after LTP induction. (control, *n* = 14 slices from nine mice; UV-irradiated, *n* = 17 slices from eight mice) (unpaired t test, ^*^P < 0.05). **h** and **i** Representative images of DCX+ and Ki-67+ cells in the DG. DCX+ cells in the DG were quantified using a graph of five mice from each group. Ki-67+ cells in the DG were quantified using a graph of six mice from each group. Scale bar, 150 µm. Mann–Whitney *U* test, ^*^P < 0.05  vs. the control group. Each bar represents the mean ± SEM of each group.

UV irradiation was performed using TL20W/12RS UV lamps (Philips, Eindhoven, Netherlands) with emission wavelengths ranging from 275–320 nm. UVC (<290 nm) wavelengths were blocked using a Kodacel filter (TA401/407; Kodak, Rochester, NY, USA) placed 2 cm in front of the UV lamp. UV intensity was measured using a UV meter (Model 585100, Waldmann, Villingen-Schwenningen, Germany). To prevent UV exposure to the eyes, the mice were shielded during anesthesia, and the dorsal skin was subjected to UV irradiation; the control group was anesthetized and sham-irradiated (Fig. 1b).

### OPR test

The OPR (object place recognition) test was performed using a modified method after 6 weeks of UV irradiation. The test setup consisted of an opaque plastic apparatus, and the procedure consisted of four phases: handling, habituation, training, and testing. Before the behavioral test, the mice were allowed to become accustomed to handling for 5 min/day for 4 days to reduce anxiety (handling phase). The mice were allowed to freely navigate the chamber without objects for 15 min to acclimatize to the environment (habituation phase). Twenty-four hours later, the mice were exposed to the two objects for 10 min (training phase). After training, one object was moved to a new location to evaluate long-term memory, and the mice were placed in the chamber and observed for 5 min (test phase). The time period when the mice began to show interest in the object at a new location was evaluated. The testing sessions were recorded using a video camera and analyzed by a blinded examiner. Object recognition was defined as the mouse’s nose touching the object. The discrimination index was calculated as the difference between the time spent exploring the novel place object and the time spent exploring the familiar object divided by the total time spent exploring both objects.

### NOR test

The NOR (novel object recognition) test was performed using a modified method after 6 weeks of UV irradiation. The handling, habituation, and training phases of the NOR test were identical to those of the OPR test. After training, one object was replaced with a new object to evaluate long-term memory, and the mice were placed in the chamber and observed for 5 min (test phase). The time period when the mice began to show interest in the new object was evaluated. The testing sessions were recorded using a video camera and analyzed by a blinded examiner. Object recognition was defined as the mouse’s nose touching the object. The discrimination index was calculated as the difference between the time spent exploring the novel place object and the time spent exploring the familiar object divided by the total time spent exploring both objects.

### Y-maze test

The Y-maze test was performed following a previously described protocol^23^. Mice were placed at the center of a Y-maze with three identical arms at a 120° angle under dim light. Movement was recorded for 7 min. Each arm was 30 cm long, 6 cm wide, and 15 cm high. The sequence of entries and total number of arm entries were recorded and counted manually in a blinded manner.

### Brain sample collection

All mice were anesthetized using isoflurane, and their brains were carefully removed. The excised brains were fixed in 4% paraformaldehyde solution overnight at 4 °C. Following fixation, the brains were immersed in a solution of 30% sucrose in 0.05 M phosphate-buffered saline (PBS). Fixed and cryopreserved brains were cut into sequential coronal sections of 35 μm thickness using a freezing microtome (Finesse E+, Thermo Shandon, Runcom, UK). The sections were stored in a cryoprotectant solution containing 25% glycerol in 0.05 M PBS at 4 °C until immunohistochemistry (IHC) was performed.

### fEPSP recording

Extracellular recordings were performed as previously described^24^. Hippocampal sagittal slices (400 μm) were prepared using a vibratome (Campden Instruments). The brain slices were incubated for 30 min in ice-cold artificial cerebrospinal fluid (120 mM NaCl, 3.5 mM KCl, 2.5 mM CaCl_2_, 1.3 mM MgSO_4_, 1.25 mM NaH_2_PO_4_, 10 mM glucose, and 26 mM NaHCO_3_, oxygenated with 95% O_2_ and 5% CO_2_). Additionally, fEPSPs (field excitatory postsynaptic potentials) from the Schaffer collateral-CA1 pathway were recorded at 31–32 °C. A stimulation intensity of 40% of the maximum response was used in the experiment. LTP (long-term potentiation) was induced using a theta burst stimulation protocol (four bursts, where each burst consisted of four pulses at 100 Hz and 200-ms interburst intervals). WinLTP software (WinLTP Ltd., Bristol, UK) was used to record and analyze the data.

### IHC

IHC was performed using the free-floating technique. Free-floating slices were incubated for 2 days at 4 °C with primary antibodies against doublecortin (DCX) (ab18723, Abcam, Cambridge, UK, 1:200) and Ki-67 (ab15580, Abcam, 1:200) in a diluent buffer comprising 1% bovine serum albumin (9048-46-8, Sigma‒Aldrich, St. Louis, MO, USA) and 1% Triton X-100 in 0.1 M phosphate buffer. After five washes with PBS, the sections were incubated for 4 h with the following secondary antibodies in dilution buffer: Alexa Fluor 594-conjugated goat anti-guinea pig IgG (A11012, Invitrogen) and Alexa Fluor 594-conjugated donkey anti-rabbit IgG (A21207, Invitrogen). After three washes with PBS, the sections were incubated with 4′,6-diamidino-2-phenylindole (2 μg/ml) for 5 min and mounted in X-CLARITY Mounting Solution (#C1310X; Logos Biosystems, Annandale, VA). Immunofluorescence images of the sections were captured using a confocal laser-scanning microscope (A1Rsi, Nikon, Tokyo, Japan).

### Instruments and analytical conditions

The samples were quantified using an ultrahigh-performance liquid chromatography‒mass spectrometry system consisting of an ExionLC system and a 6500 + QTRAP mass spectrometer with an electrospray ionization source. Data acquisition and quantification were conducted using Analyst software version 1.7. A Waters Acquity HSS T3 column (2.1 × 100 mm, 1.8 µm) was used for nontarget screening (NTs). The column and autosampler tray temperatures were set at 50 °C and 4 °C, respectively. The mobile phase consisted of 0.1% formic acid in water (A) and 5 mM ammonium formate in acetonitrile (B). The flow rate was set at 0.3 ml/min, and the injection volume was 10 μl. The mass spectrometer was optimized, and analysis was performed using multiple reaction monitoring scans in the positive and negative ion modes (Supplementary Table 1).

### Dopamine D1 and D2 receptor antagonists and dopamine treatment

Dopamine D1 receptor antagonist, 7-chloro-3-methyl-1-phenyl-1,2,4,5-tetrahydro-3-benzazepin-8-ol (SCH23390 hydrochloride, 0925, Tocris, Bristol, UK) and dopamine D2 receptor antagonist, 3,5-dichloro-N-{[(2S)-1-ethylpyrrolidin-2-yl]methyl}-2-hydroxy-6-methoxybenzamide (Raclopride, 1810, Tocris, Bristol, UK) were dissolved in 0.9% saline and administered at a dose of 0.1 mg/kg and 1 mg/kg via intraperitoneal injections in a volume of 100 μl. Dopamine hydrochloride (H8502, Merck, St. Louis, MO, USA) was dissolved in 0.9% saline and administered at doses of 1, 5, or 10 mg/kg via intraperitoneal injections in a volume of 100 μl.

### RNA-Seq

mRNA was isolated from 1 μg of total RNA using oligodT. The library was processed for 151-bp paired-end sequencing, and after mixing the samples, the library was prepared using the TruSeq stranded mRNA Sample Prep Kit (20020595, Illumina, San Diego, CA, USA). The isolated mRNA underwent fragmentation and was then utilized to synthesize single-stranded cDNA via random hexamer priming. Subsequently, the second strand was synthesized based on this template, and double-stranded cDNA was obtained. After sequentially performing end repair to create a blunt end, A-tailing, and adapter ligation, the cDNA library was amplified via PCR. The final product was confirmed using a 2100 Bioanalyzer (Agilent Technologies, Santa Clara, CA, USA). The library was quantified using the KAPA Library Quantification Kit (07960140001, Roche), and cluster generation and sequence decoding was performed using NovaSeq.

### DEG analysis

We performed RNA-Seq on UV-irradiated mice treated with or without SCH23390 and control mice. DEGs were selected for genes with an adjusted *P* value less than 0.05 and log2 (fold change) greater than 0.1 or less than −0.1.

### Gene set enrichment analysis

We performed pathway enrichment analysis for DEGs using the clusterProfiler package (v4.6.2) and Gene Ontology (GO) biological pathway terms. Significance was established using Fisher’s exact test (one-sided), followed by multiple comparisons with the Benjamini‒Hochberg method. An adjusted *P* value less than 0.05 was considered to indicate significantly enriched pathways.

### Statistics

Statistical analysis of the data from all experiments was performed using the Mann–Whitney *U* test and one-way analysis of variance (ANOVA), followed by Dunn’s post hoc test, in GraphPad Prism version 9.5.1 (GraphPad Software, San Diego, CA, USA). A *p* value less than 0.05 was considered to indicate a statistically significant difference between groups.

## Results

### Photoaged mice exhibit impaired cognitive function and neurogenesis

To investigate the effects of UV irradiation on hippocampal memory and neurogenesis, mouse skin was irradiated with UV for 6 weeks. After 6 weeks of UV irradiation, the mice underwent the OPR, NOR, and Y-maze behavioral tests, which are hippocampus-dependent behavioral tasks. We also probed whether UV radiation induces deficits in hippocampal LTP by recording fEPSPs (Fig. 1a). Clinical examination and hematoxylin and eosin staining confirmed thickening of the skin and epidermal layers, which is indicative of chronic UV exposure (Supplementary Fig. 1a, b). In the OPR behavioral test, the discrimination index was significantly lower in UV-irradiated mice than in sham-irradiated mice (Fig. 1c and Supplementary Fig. 1c, *P* = 0.0002). Similarly, in the NOR behavioral test, the discrimination index was significantly lower in UV-irradiated mice than in sham-irradiated mice (Fig. 1d, and Supplementary Fig. 1d, *P* = 0.0078). In the Y-maze behavioral test, the number of alternations was significantly lower in the UV-irradiated mice than in the sham-irradiated mice (Fig. 1e, *P* < 0.0001).

We investigated whether long-term UV irradiation caused LTP deficits by recording fEPSPs in the hippocampal CA3-CA1 Schaffer collateral pathway (Fig. 1f). Indeed, compared with sham irradiation, UV irradiation resulted in a significant LTP defect in the hippocampal CA3-CA1 region (Fig. 1f, g). To explore the impact of UV irradiation on hippocampal neurogenesis and proliferation, we analyzed biomarkers of both processes. UV irradiation significantly decreased the number of DCX-positive and Ki-67-positive neurons in the hippocampal dentate gyrus (DG) compared to sham irradiation (Fig. 1h, i). These findings demonstrated that chronic UV irradiation reduced hippocampal cognitive function and neurogenesis.

### UV exposure systemically increases dopamine levels in the peripheral and central nervous systems

**Fig. 2:**
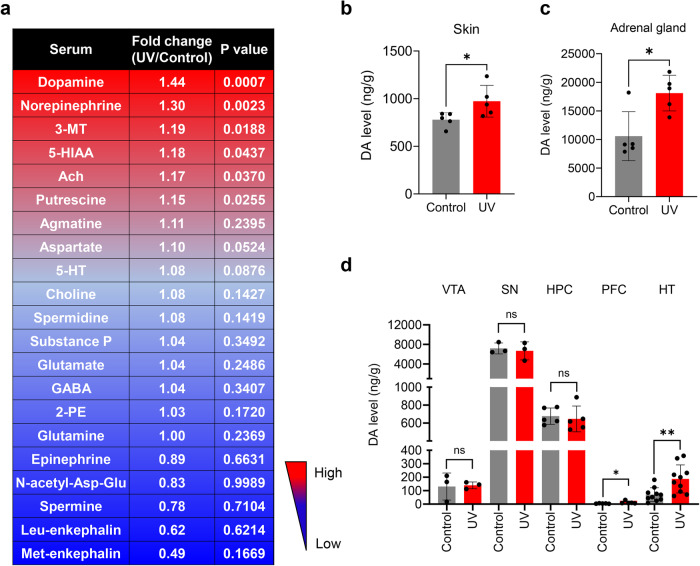
Changes in dopamine (DA) levels in peripheral organs and the central nervous system caused by UV irradiation. **a** Liquid chromatography–mass spectrometry-based neuropeptide screening revealed that DA was the most significantly increased neuropeptide in the sera of mice subjected to UV irradiation for 6 weeks. **b** Liquid chromatography–mass spectrometry-based neuropeptide screening revealed that DA levels were significantly increased in the skin of mice subjected to UV irradiation for 6 weeks. **c** Liquid chromatography–mass spectrometry-based neuropeptide screening revealed that DA was significantly increased in the adrenal glands of mice subjected to UV irradiation for 6 weeks. **d** Liquid chromatography–mass spectrometry-based neuropeptide screening revealed that DA was significantly increased in the prefrontal cortex (PFC) and hypothalamus (HT) of mice subjected to UV irradiation for 6 weeks. Mann–Whitney *U* test, ^*^P < 0.05 and ^**^P < 0.01 vs. the control group. Each bar represents the mean ± SEM of each group.

We hypothesized that UV irradiation induces the secretion of neurotransmitters from the skin, subsequently affecting brain functions. We analyzed 28 neuropeptides in mouse serum using liquid chromatography–mass spectrometry to elucidate the neurotransmitter-mediated mechanisms underlying these skin–brain interactions. Among these neuropeptides, dopamine, a catecholamine, was the most significantly upregulated. The dopamine level was ~130–145% greater in the serum of UV-irradiated mice than in that of sham-irradiated mice (Fig. 2a and Supplementary Fig. 2a). Because serum dopamine levels are influenced by the sympathetic nervous system^25^, we measured dopamine levels in the skin and adrenal glands. Dopamine levels in the skin and adrenal glands were significantly greater in UV-irradiated mice than in control mice (Fig. 2b, c). To investigate dopamine level changes in specific brain regions after UV irradiation, we quantified dopamine levels in the ventral tegmental area (VTA), substantia nigra (SN), hippocampus (HPC), prefrontal cortex (PFC), and hypothalamus (HT). In response to UV exposure, no significant changes in dopamine levels were detected in the VTA, SN, or HPC. However, dopamine levels in the PFC and HT significantly increased (Fig. 2d). These results indicate that UV irradiation increases dopamine levels in the peripheral and central nervous systems.

### Systemic blockade of the dopamine D1 receptor counteracts UV-induced memory dysfunction

**Fig. 3:**
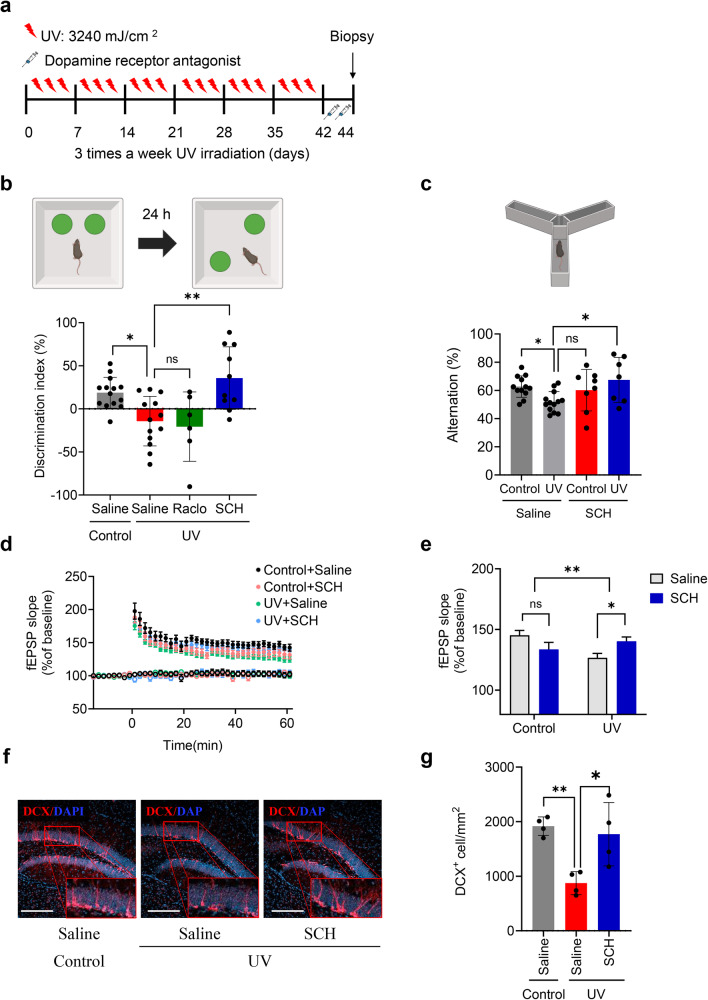
Effect of a dopamine D1 receptor antagonist on memory dysfunction after UV irradiation. **a** Overview of the experimental design. **b** Diagram representing the OPR test. The dopamine D1 receptor antagonist SCH23390 (SCH, 0.1 mg/kg, intraperitoneal injection), but not the dopamine D2 receptor antagonist raclopride (Rac, 1 mg/kg, intraperitoneal injection), rescued the memory dysfunction induced by UV irradiation. Control group (*n* = 14), UV-irradiated group (*n* = 13), raclopride-treated group (*n* = 6), and SCH23390-treated group (*n* = 10). The discrimination index was calculated by subtracting the time the mice spent exploring a familiar object (F) from the time they spent exploring a novel object (N) and dividing that difference by the total time spent exploring both objects (discrimination index = (N − F)/(N + F)). One-way ANOVA, ^*^P < 0.05, ^**^P < 0.01 vs. the control group. Each bar represents the mean ± SEM of each group. **c** Diagram representing the Y-maze test. The Y-maze test revealed that spontaneous alternation was impaired by UV irradiation. Summary of spontaneous alternations. Control group (*n* = 9), UV-irradiated group (*n* = 10), Control+SCH23390-treated group (*n* = 8), and UV + SCH23390-treated group (*n* = 7). The percentage of alternations in the Y-maze was significantly greater than the chance level in the control group but not in the UV-irradiated group. SCH (0.1 mg/kg) rescued memory dysfunction in UV-irradiated mice. One-way ANOVA, ^*^P < 0.05 vs. the control group. Each bar represents the mean ± SEM of each group. One-way ANOVA, ^*^P < 0.05 vs. the control group. Each bar represents the mean ± SEM of each group. **d** LTP was analyzed by the time course of the fEPSP slope and was induced by 4X theta burst stimulation (4XTBS, each burst with four stimuli at 100 Hz, 200 ms interburst interval) in hippocampal slices of four groups of mice. **e** Average fEPSP slope 51–60 min after LTP induction. Hippocampal LTP deficits in the 6-week UV-treated mice were restored by dopamine D1 receptor antagonist delivery (saline-injected control, *n* = 8 slices from 6 mice; SCH-injected control, *n* = 9 slices from 5 mice; saline-injected UV-irradiated, *n* = 18 slices from 11 mice; SCH-injected UV-irradiated, *n* = 13 slices from 10 mice; two-way ANOVA, effect of interaction between UV treatment and drug treatment, ^**^P < 0.01, Sidak’s multiple comparison Saline-UV vs. SCH-UV, ^*^P < 0.05). **f** Representative images of DCX+ cells in the DG. **g** DCX+ cells in the DG were quantified in five mice from each group. Scale bar, 150 µm. One-way ANOVA, ^*^P < 0.05, ^**^P < 0.01 vs. the control group. Each bar represents the mean ± SEM of each group.

To determine whether elevated dopamine levels contributed to cellular and behavioral deficits in UV-irradiated mice, we administered intraperitoneal injections of either SCH23390 (0.1 mg/kg, 100 μl, dopamine D1 receptor antagonist) or raclopride (dopamine D2 receptor antagonist, 1 mg/kg, 100 μl) to UV- or sham-irradiated mice (Fig. 3a). In the OPR test, the discrimination index was significantly lower in UV-irradiated mice injected with vehicle (saline) than in sham-irradiated mice. While the discrimination indices did not significantly change in the raclopride-treated group, the discrimination indices of the SCH23390-treated group were significantly greater than those of the UV-irradiated group (Fig. 3b and Supplementary Fig. 3a). In the Y-maze test, the number of alternations was also significantly lower in UV-irradiated mice and significantly greater in the SCH23390-treated group than in the UV-irradiated group (Fig. 3c). LTP was significantly impaired in the 6-week UV-treated group compared to the control group, in line with prior LTP data (Fig. 1f, g). Consistent with the behavioral test results, LTP was significantly greater in the 6-week UV-irradiated mice treated with SCH23390 than in the vehicle-treated UV-irradiated mice. LTP in the control sham group treated with SCH23390 did not significantly differ from that in the control group treated with saline. These findings indicate that systemic injections of a dopamine D1 receptor antagonist can restore hippocampal LTP in 6-week UV-treated mice (Fig. 3d, e).

To explore the neuroprotective effects of dopamine D1 receptor antagonists in UV-irradiated mice, we analyzed biomarkers of neurogenesis. UV-irradiated mice treated with SCH23390 (UV-SCH) exhibited significantly greater numbers of DCX-positive neurons in the hippocampal DG than did UV-irradiated saline-injected mice (Fig. 3f, g). These results imply that the dopamine D1 receptor antagonist SCH23390 could improve hippocampal memory and synaptic plasticity and confer neuroprotection against memory dysfunction induced by UV irradiation.

### Chronic UV irradiation induces transcriptomic changes linked to the dopaminergic neuron differentiation pathway

**Fig. 4:**
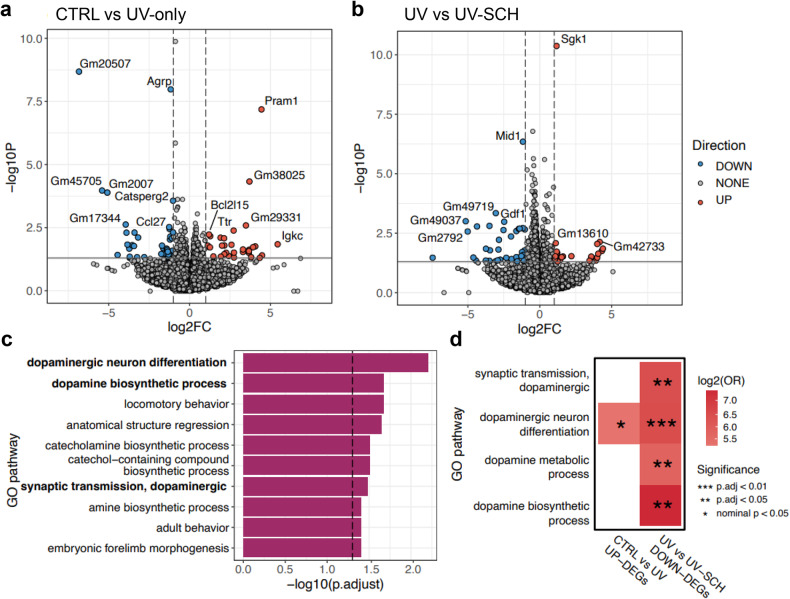
SCH23390 treatment after UV irradiation suppresses dopaminergic neuronal differentiation. **a** Volcano plot of DEGs between the control and UV treatment groups. Genes that were significantly downregulated and upregulated are shown in blue and red, respectively. **b** Volcano plot of DEGs between UV-irradiated mice and UV-SCH-treated mice. Genes that were significantly downregulated and upregulated are shown in blue and red, respectively. **c** Top significant GO biological pathways for downregulated DEGs in UV-irradiated mice vs. UV-SCH-irradiated mice. Dopamine-related pathways are marked in bold letters. **d** Enrichment analysis results for upregulated and downregulated DEGs involved in dopamine-related GO pathways in control vs. UV-irradiated mice and in UV-irradiated mice vs. UV-SCH, respectively. The color represents the log2 (odds ratio) value, and significance is indicated by asterisks.

Our findings indicate that antagonizing dopamine signaling ameliorated memory dysfunction in the hypothalamus. To elucidate the transcriptomic changes following UV irradiation and SCH23390 treatment, we conducted RNA sequencing (RNA-Seq) analysis of the DEGs. Seventy DEGs were significantly altered in UV-irradiated mice compared to sham-irradiated controls (Fig. 4a). Compared with saline treatment, UV-SCH treatment resulted in 38 notably downregulated genes (Fig. 4b). By conducting a GO enrichment analysis, we found that the downregulated DEGs in the UV-SCH group were predominantly enriched in dopamine-related pathways compared to those in the UV-irradiated group (Fig. 4c). Specifically, genes such as *Pax5, Foxa2*, and *En1* were most profoundly enriched within the dopaminergic neuron differentiation pathway. This pathway was nominally enriched in the DEGs between the SCH23390-treated group and the control group (Fig. 4d). These findings suggest that the dopaminergic neuronal differentiation induced by UV irradiation might be reversed by SCH23390 treatment.

### Chronic peripheral dopamine injections impair hippocampal memory and neurogenesis

**Fig. 5:**
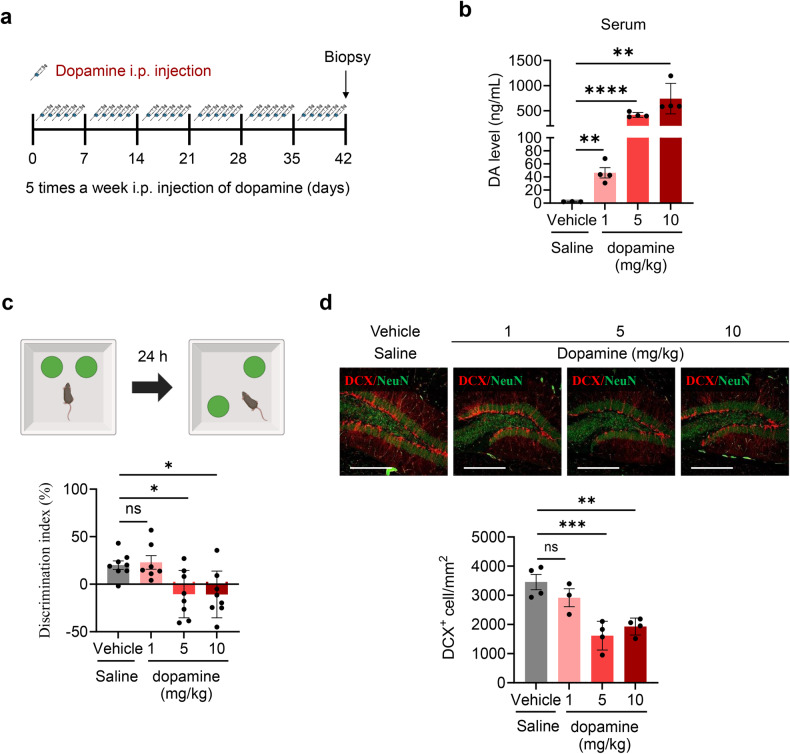
Effects of dopamine (DA) injection on memory function. **a** Overview of the experimental design. **b** Liquid chromatography–mass spectrometry-based analysis of DA levels in serum after intraperitoneal injection of DA. One-way ANOVA, ^**^P < 0.01, ^****^P < 0.0001 vs. the saline group. Each bar represents the mean ± SEM of each group. **c** Diagram of the obje**c**t place recognition test. Vehicle (*n* = 8); dopamine 1 mg/kg group (*n* = 7); dopamine 5 mg/kg group (*n* = 8); dopamine 10 mg/kg group (*n* = 8). The discrimination index was calculated by subtracting the time the mice spent exploring a familiar object (F) from the time they spent exploring a novel object (N) and then dividing that difference by the total time spent exploring both objects (discrimination index = (N − F)/(N + F)). One-way ANOVA, ^*^P < 0.05 vs. the saline group. Each bar represents the mean ± SEM of each group. **d** Representative images of DCX+ cells in the DG. DCX+ cells in the DG were quantified using a graph of four mice from each group. Scale bar, 150 µm. One-way ANOVA, ^**^P < 0.01 and ^*^^*^^*^P < 0.001 vs. the saline group. Each bar represents the mean ± SEM of each group.

Our results strongly suggest that the increase in dopamine levels may be responsible for the UV irradiation-induced deficits in brain functions. To examine the effects of peripheral dopamine on hippocampal memory and neurogenesis, mice were intraperitoneally injected with either saline or dopamine (1, 5, or 10 mg/kg, 100 μl) for 6 weeks (Fig. 5a). After dopamine injection, serum dopamine levels significantly increased in a concentration-dependent manner (Fig. 5b). Six weeks postinjection, the mice were subjected to the OPR test. Mice treated with dopamine (5 and 10 mg/kg) exhibited a lower discrimination index than those treated with saline. (Fig. 5c and Supplementary Fig. 4a). To evaluate the influence of peripheral dopamine injections on hippocampal neurogenesis, neurogenesis biomarkers were analyzed. Dopamine injections (at 5 and 10 mg/kg) over 6 weeks substantially reduced the number of DCX-positive cells in the hippocampal DG compared to that in the saline-treated group (Fig. 5d). These findings highlight that prolonged dopamine injections, specifically at 5 and 10 mg/kg doses, could compromise hippocampal cognitive function and neurogenesis, reproducing some cognitive alterations observed with UV exposure.

## Discussion

**Fig. 6:**
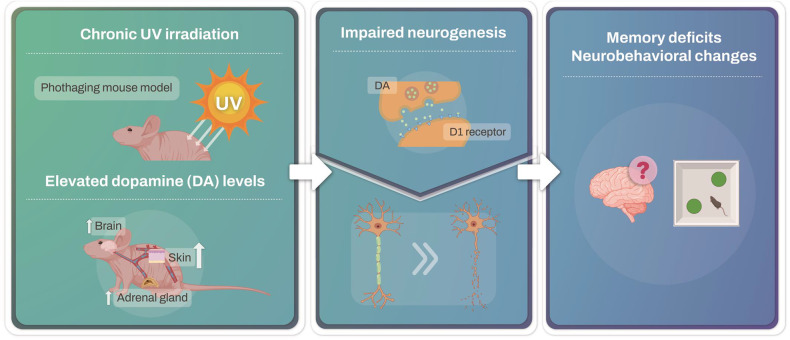
UV exposure activates the dopamine D1 receptor signaling pathway. UV irradiation elevates dopamine levels in the skin, adrenal glands, and brain. Elevated dopamine levels lead to activation of the dopamine D1 receptor signaling pathway. UV exposure-induced activation of the dopamine D1 receptor signaling pathway induces memory deficits and neurobehavioral changes.

We revealed an intriguing link between chronic UV irradiation, dopamine D1/D5 receptor signaling, and cognitive impairment. Our data indicate that (1) repeated UV irradiation can lead to cognitive deficits and reduced neurogenesis; (2) chronic UV exposure elevates dopamine levels in the skin, adrenal glands, and brain; (3) UV exposure causes cognitive alterations by acting through the dopamine D1 receptor; and (4) the administration of dopamine in mice replicates the cognitive impacts observed with UV exposure. Central to our findings, we determined that the activation of the dopamine D1 receptor signaling pathway by UV exposure is pivotal for modulating neurogenesis, synaptic plasticity, and cognitive function (Fig. 6).

The intricate mechanism underlying memory dysfunction via dopamine signaling involves complex interactions within the brain’s neural circuits^26^. Dopamine influences multiple facets of memory formation and consolidation, primarily through its effects on synaptic plasticity, which is the ability of synapses to strengthen or weaken based on activity^20^. While dopamine is typically associated with enhanced learning and memory by strengthening synaptic connections^27^, excessive dopamine release can have detrimental effects on synaptic plasticity, including LTP^28,29^. Additionally, although dopamine signaling is crucial for various cognitive functions, excessive activation of dopamine D1/D5 receptors can interfere with LTP^30^ and induce cytotoxicity^31^. We demonstrated that repeated UV exposure increased dopamine levels in both peripheral organs and the brain. This surge might contribute to cognitive deficits, and blocking dopamine signaling with a dopamine D1 receptor antagonist could ameliorate these memory deficits induced by UV irradiation. Our findings provide compelling evidence that chronic UV exposure causes cognitive impairment linked to changes in dopamine levels and dopamine D1 receptor activity.

Although dopamine levels can be influenced by a wide range of factors, including drugs, stress, and certain medical conditions^32^, there has been limited evidence to suggest that exposure to UV irradiation directly causes a significant increase in dopamine levels^33^. Exposure to UV radiation can have several indirect effects on dopamine and other neurotransmitters. Previous studies have suggested a connection between vitamin D levels and mood disorders^34^ and that dopamine circuits can be modulated by vitamin D signaling. Increased vitamin D production due to sunlight exposure might indirectly affect dopamine levels^35^.

Upregulated components of signaling pathways related to dopaminergic neuronal differentiation can potentially have both advantageous and detrimental effects on memory, depending on various factors, including context, timing, and degree of elevation^21,36^. Our transcriptomic analysis revealed specific enrichment in dopaminergic neuron differentiation pathways associated with UV exposure and SCH23390 treatment. This enrichment indicates the involvement of dopamine-related signaling pathways in mediating the neurobehavioral effects in response to UV exposure. These results deepen our understanding of how UV radiation can influence brain function and behavior, potentially through its impact on dopamine signaling.

Dopamine levels and cognitive function exhibit an inverted U-shaped relationship, suggesting that cognitive performance can be reduced with either deficient or excessive dopamine, whereas peak cognitive function is associated with an optimal dopamine level^37^. Insufficient dopamine can lead to difficulty maintaining attention, reduced motivation, and impaired working memory^32,38^. Conversely, excessive dopamine can impair cognitive function^39^, inducing impulsivity, distractibility, and difficulties in maintaining a stable focus, as noted in conditions such as schizophrenia, where dopamine pathways are hyperactive^40,41^. We showed that sustained peripheral dopamine injections, particularly at higher doses, impaired hippocampal memory and neurogenesis. This finding highlights the importance of dopamine regulation for cognitive health. In addition, peripheral manipulation of dopamine can replicate the cognitive alterations caused by UV exposure, underscoring the intertwined connection between these two factors in modulating cognitive performance.

The absence of increased dopamine levels in the hippocampus led us to contemplate several possible explanations. First, it is plausible that elevated dopamine levels in regions such as the hypothalamus or cortex, rather than the hippocampus, may primarily influence hippocampal function. This highlights the intricate interplay among different brain regions in regulating cognitive processes^42^. Second, while dopamine levels might have increased in the hippocampus, the change could have been too subtle to be detected by our methodologies, suggesting potential limitations in the sensitivity of our measurement techniques^43^. The nuances in dopamine dynamics in the brain warrant further exploration to comprehensively understand their impact on cognitive performance and the specific mechanisms involved. The inverted U-shaped relationship between dopamine and cognitive function remains a critical consideration in this context, emphasizing the importance of precise dopamine regulation for optimal cognitive health. In conclusion, this investigation underscores the necessity for UV protection, and further research is needed to fully understand the intricate mechanisms through which UV exposure affects brain function and neurotransmitter systems. This study also highlights the potential of pharmacological interventions targeting dopamine receptors to mitigate the negative neurological outcomes of UV exposure.

### Limitations and suggestions for further study

UV exposure may influence brain function in multiple ways. Further research is needed to fully understand the underlying mechanisms and potential therapeutic applications involved. Individual responses to UV exposure may vary in humans, and caution should be exercised when translating and applying the findings of animal studies to humans. Additionally, the prospect of pharmacological interventions targeting dopamine receptors, as highlighted in this study, mandates in-depth research to determine their safety and effectiveness in humans.

### Supplementary information

Supplementary Information

## Data Availability

All data generated or analyzed during this study are included in this article and its supplementary information files.
