# Supplementary material for: Chronic ultraviolet irradiation induces memory deficits via dysregulation of the dopamine pathway
Source: Exp Mol Med. 2024 Jun 3;56(6):1401–11. doi: 10.1038/s12276-024-01242-x (PMC11263540; doi:10.1038/s12276-024-01242-x)
Supplement: Supplementary file 1 — Supplementary Information [file 12276_2024_1242_MOESM1_ESM.pdf]

Supplementary Figures and Table

Supplementary Table 1. MRM transitions, retention times, and other conditions of each analyte and internal standard

Analyte	Q1 Mass (*m/z*)	Q3 Mass (*m/z*)	CE (V)	CXP (V)	Retention time (min)	Ionization polarity
GABA	104.0	87.0	15.00	10.00	1.0	Positive
Glutamate	148.0	84.2	25.00	10.00	0.9	Positive
5-HT	177.1	160.1	21	18	3.2	Positive
5-HIAA	192.0	146.1	23	16	3.5	Positive
Norepinephrine (NE)	170.0	152.1	12	16	1.1	Positive
MHPG-sulfate	263.0	165.1	-20	-9	3.1	Negative
Acetylcholine	146.9	88.1	21	10	1.0	Positive
Choline	104.1	60.0	20	10	0.9	Positive
DA	154.0	137.0	21.00	16.00	1.3	Positive
DOPAC	167.0	123.0	-14	-7	3.5	Negative
HVA	180.9	136.9	-12	-15	3.6	Negative
3-MT	168.1	151.0	13	16	3.2	Positive
Tyramine	138.0	121.1	13	14	3.2	Positive
Tryptamine	161.0	144.0	13	14	3.5	Positive
Octopamine	154.0	136.0	11	6	1.4	Positive
2-phenethylamine	122.0	105.1	15	12	3.4	Positive
Dynorphin A	491.3	434.8	29	12	3.5	Positive
Leu-enkephalin	556.2	120.0	73	12	3.6	Positive
Met-enkephalin	574.2	120.0	77	14	3.6	Positive
Substance P	674.0	594.0	39	8	3.5	Positive
Aspartic acid	134.0	74.0	19	8	0.8	Positive
Epinephrine	184.1	166.0	10	20	1.3	Positive
Glutamine	147.0	84.0	18	10	0.9	Positive
N-acetyl-Asp-Glu	305.1	148.0	15	16	3.1	Positive
Agmatine	131.0	72.1	21	8	1.0	Positive
PTR	89.4	72.0	17	8	0.8	Positive
Spermidine	146.0	72.0	15	8	0.9	Positive
Spermine	203.1	129.1	15	8	0.8	Positive

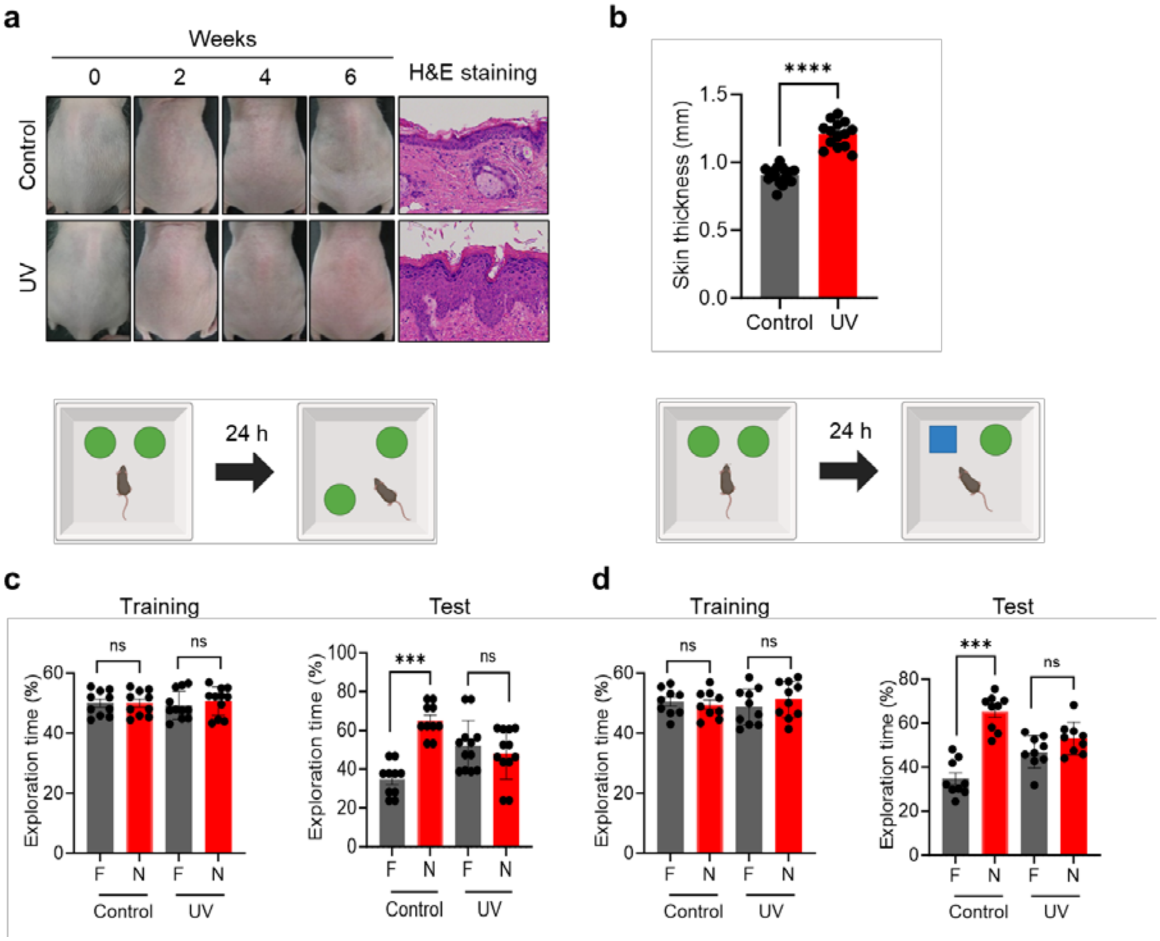

Supplementary Fig. 1. Effects of UV irradiation on memory function. a) Changes in skin image and hematoxylin and eosin staining after 6 weeks of UV irradiation. **b)** Relative skin thickness changes in control and UV-irradiated mice. c**)** OPR tests. Mice were exposed to two identical objects in a square box for training, and one object was moved to a different location after 24 h. The control group mice (n = 10) could differentiate between objects that were moved and those that were not, whereas UV-irradiated mice (n = 11) could not. Paired two-tailed t-test, ***$P < 0.001$ (familiar vs. novel in the control group) and $P = 0.6236$ (familiar vs. novel in the UV group). **d)** NOR test. Mice were exposed to two identical objects in a square box for training, and one object was replaced with a new object after 24 h. The control group mice (n = 9) could differentiate novel objects from familiar objects, whereas the UV-irradiated mice (n = 9) could not. Paired two-tailed t-

test, ***P < 0.001 (familiar vs. novel in the control group) and P = 0.2488 (familiar vs. novel in the UV group).

a

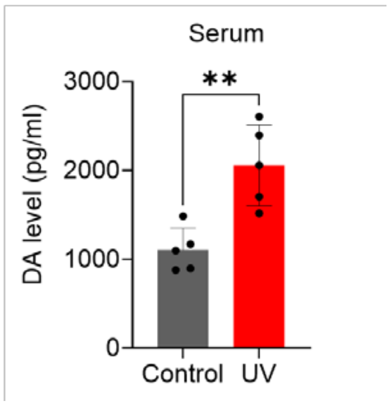

Supplementary Fig. 2. Changes in dopamine (DA) level by UV irradiation. a) ELISA was used to determine DA levels. DA level was significantly upregulated in the serum of mice after 6 weeks of UV irradiation. **$P < 0.01$ vs. control group. Each bar represents the mean±SEM of each group.

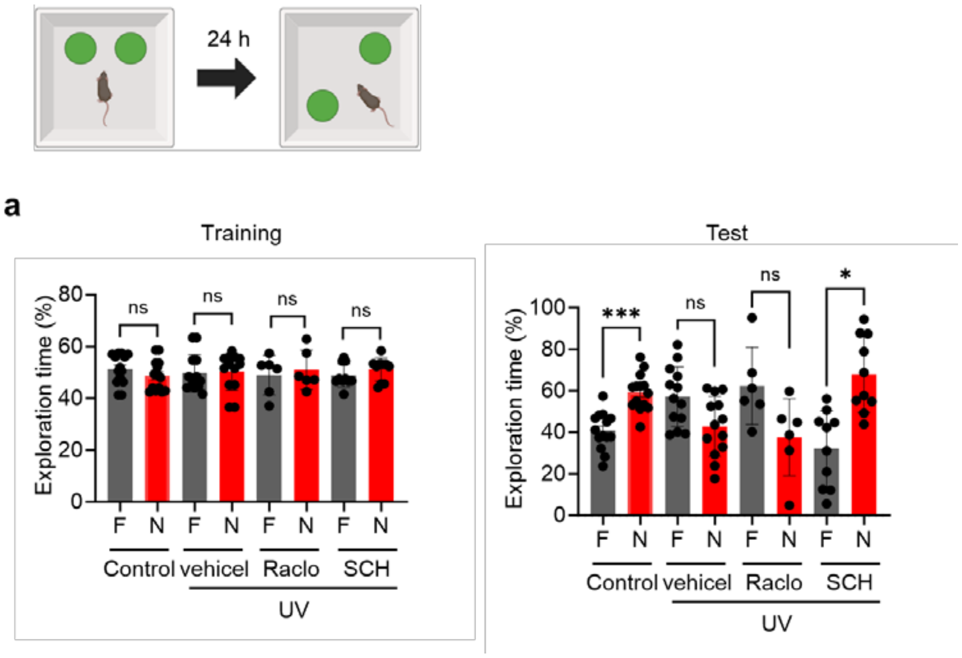

Supplementary Fig. 3. Effect of dopamine D1 receptor antagonist, SCH23390, on memory dysfunction by UV irradiation. a) OPR tests. The mice were exposed to two identical objects in a square box for training, and one object was moved to a different location after 24 h. The control group mice (n = 14) and SCH23390-treated mice (n = 10) could differentiate between objects that were moved and those that were not, whereas UV-irradiated mice (n = 11) and raclopride-treated mice (n = 6) could not. Paired two-tailed t-test, ****P* < 0.001 (familiar vs. novel in the control group), *P* = 0.0982 (familiar vs. novel in UV group), *P* = 0.1656 (familiar vs. novel in the raclopride-treated group), and *P* < 0.05 (familiar vs. novel in the SCH23390-treated group).

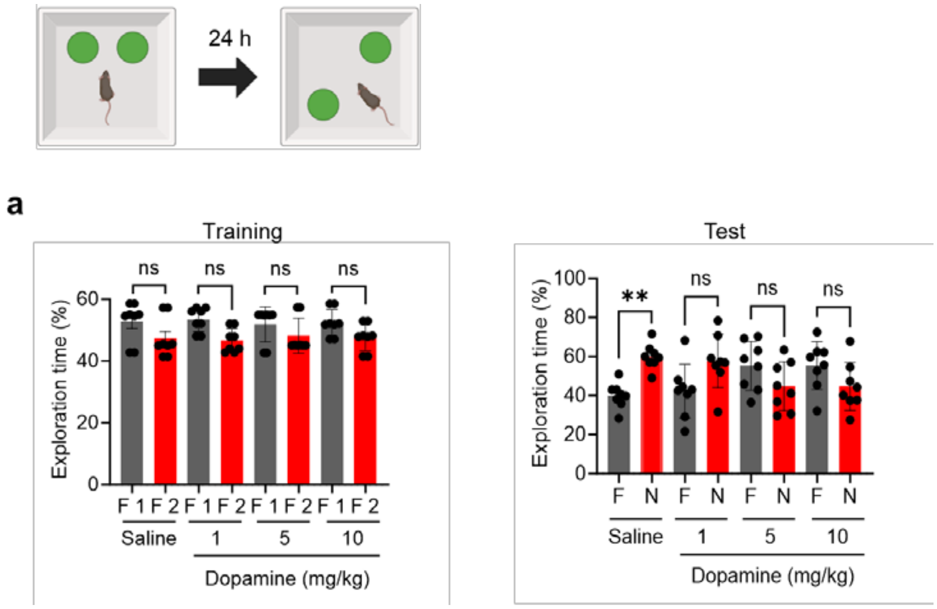

Supplementary Fig. 4. Effects of dopamine injection on memory function. a) OPR tests. The mice were exposed to two identical objects in a square box for training, and one object was moved to a different location after 24 h. The saline group mice (n = 8) could differentiate between objects that were moved and those that were not, whereas dopamine 1mg/kg-treated mice (n = 8), dopamine 5mg/kg-treated mice (n = 8), and dopamine 10mg/kg-treated mice (n = 8) could not. Paired two-tailed t-tests, $^{**}P < 0.01$ (familiar vs. novel in the saline group) $P = 0.1545$ (familiar vs. novel in the dopamine 1mg/kg-treated mice), $P = 0.2759$ (familiar vs. novel in the dopamine 5mg/kg-treated mice), and $P = 0.2557$ (familiar vs. novel in the dopamine 10mg/kg-treated mice).
